# The buffering effect of tangible social support on financial stress: influence on psychological well-being and psychosomatic symptoms in a large sample of the adult general population

**DOI:** 10.1186/s12939-014-0085-3

**Published:** 2014-09-28

**Authors:** Cecilia Åslund, Peter Larm, Bengt Starrin, Kent W Nilsson

**Affiliations:** Centre for Clinical Research Västerås, Uppsala University, Västmanland County Hospital Västerås, Västerås, Sweden; School of Health, Care, and Social Welfare, Mälardalen University, Västerås, Sweden; Department for Social Studies, Karlstad University, Karlstad, Sweden

**Keywords:** Buffering effect, Economic stress, Public health, Self-rated health, Social support

## Abstract

**Introduction:**

Financial stress is an important source of distress and is related to poor mental and physical health outcomes. The present study investigated whether tangible social support could buffer the effect of financial stress on psychological and psychosomatic health.

**Methods:**

Two separate postal surveys were sent to random samples in five counties in Sweden in 2004 and 2008, with a total of 84 263 respondents. The questionnaires included questions about financial stress, tangible social support, psychosomatic symptoms, and psychological well-being (General Health Questionnaire-12).

**Results:**

Individuals with high financial stress and low tangible social support had six to seven times increased odds ratios for low psychological well-being and many psychosomatic symptoms. By contrast, individuals with high financial stress and high tangible social support had only two to three times increased odds ratios for low psychological well-being and three to four times increased odds ratios for many psychosomatic symptoms, suggesting a buffering effect of tangible social support. Consistent with the buffering hypothesis, there were significant interactions between financial stress and social support, particularly in relation to low psychological well-being.

**Conclusions:**

Social support had its strongest effect at high levels of financial stress. The question whether the altering of our social networks may improve physical health is important for the prevention of ill health in people experiencing financial stress. Strengthening social networks may have the potential to influence health-care costs and improve quality of life.

## Background

Financial stress, the persistent inability to afford the basic necessities of life, is an important source of distress and is related to poor mental and physical health outcomes in the population [1-4]. Financial stress has been suggested to be a powerful independent predictor for both the onset and persistence of episodes of mental disorders [5]. A common health-promoting factor often discussed in relation to chronic life strain is social support. It refers to the material and emotional resources that are available to a person through interpersonal contacts. It is associated with a reduced risk of a wide range of health outcomes including mental health [6-9], physical health [6,7,9-11], and mortality [6,9,12,13].

Social support can be assessed by both community-centric and person-centric instruments. There is an ongoing debate about whether social support enhances health irrespective of the level of stress (main or direct effect) or because social support buffers, or moderates, the effects of stress (interactive effect) [14-16]. The existence of a buffering effect requires a statistically significant interaction between the measures of social support and stress [16]. Stress-buffering effects have been observed inconsistently [15,17], whereas the main effect of social support on health has generally been replicated [18]. It has been suggested that the buffering effect on a specific life stress is observed only for social support aimed at alleviating the specific stress [14,15] and that tangible social support is the most probable aspect of social support that can buffer the effects of financial stress [14]. Peirce et al. suggested that research on social support and health should focus on specific dimensions of perceived support in combination with carefully matched types of life stress [19]. Although many studies have investigated the buffering effect of different aspects of social support on general life stress, few studies have focused on the relationship between financial stress and tangible social support in the context of the suggestions mentioned above.

The present study followed these suggestions by investigating the buffering effect of tangible social support on financial stress in relation to low psychological well-being and psychosomatic symptoms. The hypothesis was that high tangible social support would buffer the effects of financial stress on psychological and psychosomatic health. By investigating these specific associations in a large (N > 80 000) sample of the adult general population, the study contributes important new information to the research field.

## Methods

### Study design, population and procedure

This was a cross-sectional study. Postal surveys are distributed every four years by the county councils in the five Swedish counties of Uppsala, Sörmland, Västmanland, Värmland, and Örebro, in order to monitor the psychosocial health of the population. In total, the five counties comprise about 1 400 000 inhabitants. The present study involves data from one survey that was distributed during September to November 2004, and one survey that was distributed during March to May 2008. Random samples of 68 460 people for the 2004 survey and 68 710 for the 2008 survey, aged 18–84 years and stratified by sex, age, and city (and parts of the city for larger cities), were drawn from the total population by Statistics Sweden. They were asked to participate in each study by completing a postal survey questionnaire supplied with a prepaid return envelope. After 10 days, a reminder was sent to the participants who had not responded. Ten days after the first reminder, a new questionnaire was sent together with a second reminder. A new questionnaire together with a third reminder was sent four weeks after the first reminder. The questionnaires were scanned and transformed into a data file with no personal identification of the participants.

The study followed the Swedish guidelines for studies of social sciences and humanities according to the Declaration of Helsinki. According to Swedish law (Ethical Review Act 2003:460), this type of anonymous study does not require ethical approval by a medical faculty.

### Measures

#### Demographic data

Sex was categorised as (1) male and (2) female. Age was categorised as (1) 18–24 years, (2) 25–34 years, (3) 35–44 years, (4) 45–54 years, (5) 55–64 years, (6) 65–74 years, and (7) 75–84 years. Employment status was categorised as (1) employed, (2) self-employed, (3) on parental leave, (4) on leave, other reasons (5) full-time housewife/husband, (6) student, (7) unemployed, (8) on sick leave or early retirement, (9) old-age pension, and (10) other. Information about country of birth was obtained for each participant from Statistics Sweden and classified as (1) Sweden, (2) other country in Scandinavia, and (3) country outside of Scandinavia.

#### Year of survey

Societal factors such as trade conditions and unemployment rates might have changed between the two surveys. Such societal factors might be associated with both financial stress and ill health and could affect the results. For example, it has been shown that the association between unemployment and mortality weakens as the general unemployment rate increases [20]. Therefore, we adjusted all analyses for year of survey.

#### Psychosomatic symptoms

The questionnaire asked the question “How often during the past three months have you experienced the following symptoms: (i) pain in the shoulders/neck; (ii) pain in the back/hips; (iii) pain in the hands/arms/legs/knees/feet; (iv) abdominal pain; (v) headache/migraine; (vi) anxiety/nervousness; (vii) feelings of fatigue/feebleness; (viii) sleeping problems; (ix) depression; (x) dizziness; or (xi) irritated mucous membranes?” The responses options were: 0, never; 1, rarely; 2, several times; and 3, most of the time. The internal consistency of the questions of psychosomatic symptoms was α = 0.858. A summation index was created with a range of 0–36 points. The index was divided by the SD and was dichotomised with +1 SD as the cut-off point for having many psychosomatic symptoms.

#### Psychological well-being

The short version of the General Health Questionnaire (GHQ-12) was used. In this study, the Goldberg GHQ scoring method was applied [21], in which the responders can score 0-0-1-1 to give a total sum of 0–12 points. The internal consistency of the GHQ-12 items was α = 0.913. A summation index was created; for dichotomisation, a score of ≥3 was categorised as reduced psychological well-being [22].

#### Other chronic disease

The participants were asked: “Have you experienced any long-lasting disease (>6 months), any persistent symptoms following an accident, any disability, or any other long-lasting health problem?”. The response options were: 1, no; and 2, yes.

#### Financial stress

This measure was adapted according to previous measurements of financial stress [23], using the following two questions.

1. “During the past 12 months, has it been difficult for you to pay your monthly costs, such as rent, mortgage, etc.?” The response options were: no; yes, one month; yes, 2 months; yes, 3–5 months; and yes, 6–12 months. No economic difficulties was scored as 0 points, and economic difficulties for ≥1 month was scored as 1 point.

2. “If you suddenly ended up in an unforeseen situation where you had to raise SEK 20 000 (~€ 2100), would you be able to?” The response options were: yes (0 points) or no (1 point).

The variable financial stress was created by summing the points for questions 1 and 2, and then categorising the result as: no financial stress, 0 points (no difficulties with monthly costs and able to raise money); medium financial stress, 1 point (either difficulties with monthly costs or unable to raise money); and high financial stress, 2 points (difficulties with monthly costs and unable to raise money).

#### Tangible social support

This measure was adapted according to previous measurements of tangible or instrumental social support [24,25]. Tangible social support was measured by three questions as follows. “Do you have persons around you who would give you support in the event of personal problems or crises?” “Do you have persons around you who would help you with grocery shopping/cooking if you should fall ill?” “Do you have persons around you who would help you if you were to move to another dwelling?” The response options were: 1, no; 2, probably not; 3, yes, probably; and 4, yes, definitely. The internal consistency of the questions on tangible social support was α = 0.769. A summation index was created with a range of 4–12 points. The index was divided by the SD and dichotomised with −1 SD as the cut-off point for having low tangible social support.

#### Operationalisation of financial stress — tangible social support model

We created a six-quadrant model by combining the three levels of financial stress (no financial stress, medium financial stress, and high financial stress) with the two levels of tangible social support (low and high tangible social support).

#### Statistical analyses

Missing data were addressed by complete case analysis. The potential confounding variables of age, country of birth, employment status, other chronic disease, and year of the survey were adjusted for in the analyses. Because of a large number of missing cases (about 13%), educational level was not included as a control variable in the analyses.

Sex differences in the dependent and independent variables were analysed by χ^2^ test. The six-quadrant model was analysed using two separate categorical binary logistic regression models adjusted for age, country of birth, employment status, chronic disease, and year of survey to investigate any associations with psychological well-being and psychosomatic symptoms. All analyses were separated by sex. Differences between the subgroups in the six-quadrant model in relation to the outcome variables were analysed by χ^2^ test. Interaction effects between financial stress and tangible social support in relation to reduced psychological well-being and psychosomatic symptoms were analysed by general linear model (GLM).

All statistical analyses were performed using the Statistical Package for the Social Sciences for Windows, version 20.0.

## Results

In total, there were 84 263 respondents, of which 45 704 (54.2%) were women and 38 559 (45.8%) were men (p < 0.001). The response rates were 63.7% for the 2004 survey and 59.2% for the 2008 survey, giving a combined response rate 61.4%. The descriptive data for the response rates according to county, sex, age, education, country of birth, and educational level are described in detail in the articles by Nilsson et al. [26] for the 2004 survey and by Åslund et al. [27] for the 2008 survey. The mean ages were 52.8 years (standard deviation, SD = 18.3) for the 2004 survey and 53.8 years (SD = 17.9) for the 2008 survey.

As shown in Table 1, financial stress was more common among women, and low tangible social support was more common among men. Compared with men, a higher percentage of women reported low psychological well-being and many psychosomatic symptoms.

**Table 1:** **Description of the study participants, showing analysis of sex differences by χ** ^**2**^

	**Total**		**Men**		**Women**			
	**N**	**%**	**N**	**%**	**N**	**%**	**χ** ^**2**^	**p**
*Year of survey*								
2004	43 589	51.7	20 060	52.0	23 529	51.5	276.08	<0.001
2008	40 674	48.3	18 499	48.0	22 175	48.5	332.23	<0.001
*Age, years*								
18–24	6630	7.9	2665	6.9	3965	8.7	254.90	<0.001
25–34	9958	11.8	4030	10.5	5928	13.0	361.76	<0.001
35–44	11 488	13.6	4975	12.9	6513	14.3	205.91	<0.001
45–54	12 630	15.0	5494	14.2	7136	15.6	213.47	<0.001
55–64	15 482	18.4	7336	19.0	8146	17.8	42.38	<0.001
65–74	16 974	20.1	8769	22.7	8205	18.0	18.74	<0.001
75–84	11 101	13.2	5290	13.7	5811	12.7	24.45	<0.001
*Country of birth*								
Sweden	74 659	88.6	34 307	89.0	40 352	88.3	489.45	<0.001
Other country in Scandinavia	4431	5.3	1848	4.8	2583	5.7	121.92	<0.001
Country outside of Scandinavia	5173	6.1	2404	6.2	2769	6.1	25.75	<0.001
*Employment status*								
Employed	34 349	40.8	15 815	41.0	18 534	40.6	215.23	<0.001
Self-employed	4428	5.4	3089	8.0	1339	2.9	691.62	<0.001
On parental leave	1471	1.7	144	0.4	1327	2.9	951.39	<0.001
On leave, other reasons	85	0.1	34	0.1	51	0.1	3.40	0.065
Full-time housewife/husband	752	0.9	93	0.2	659	1.4	426.01	<0.001
Student	4239	5.0	1458	3.8.	2781	6.1	412.91	<0.001
Unemployed	2831	3.4	1172	3.0	1659	3.6	83.78	<0.001
On sick leave/early retirement	4809	5.7	1675	4.3	3134	6.9	442.65	<0.001
Old-age pension	25 702	30.5	13 048	33.8	12 654	27.7	6.04	0.014
Other	3023	3.6	1091	2.8	1932	4.2	233.97	<0.001
Missing data	2574	3.1	940	2.4	1634	3.6	187.12	<0.001
*Other chronic disease*								
Yes	25 836	30.7	11 525	29.9	14 311	31.3	300.43	<0.001
No	55 269	65.6	25 645	66.5	29 624	64.8	286.46	<0.001
Missing data	3158	3.7	1389	3.6	1769	3.9	45.73	<0.001
*Financial stress*								
No financial stress	59 348	70.4	29 026	75.3	30 322	66.3	28.30	<0.001
Medium financial stress	15 740	18.7	6015	15.6	9725	21.3	874.47	<0.001
High financial stress	6758	8.0	2525	6.5	4233	9.3	431.68	<0.001
Missing data	2417	2.9	993	2.6	1424	3.1	76.86	<0.001
*Tangible social support*								
High social support	70 757	84.0	31 760	82.4	38 997	85.3	740.20	<0.001
Low social support	11 793	14.0	6007	15.6	5786	12.7	4.14	0.042
Missing data	1713	2.0	792	2.1	921	2.0	9.72	0.002
*Psychological well-being*								
Normal/high	70 218	83.3	33 639	87.2	36 579	80.0	123.10	<0.001
Reduced	13 465	16.0	4648	12.1	8817	19.3	1290.80	<0.001
Missing data	580	0.7	272	0.7	308	0.7	2.23	0.135
*Psychosomatic symptoms*								
Few-medium symptoms	69 582	82.6	34 242	88.8	35 340	77.3	17.33	<0.001
Many symptoms	13 925	16.5	4000	10.4	9925	21.7	2521.05	<0.001
Missing data	756	0.9	317	0.8	439	1.0	19.69	<0.001
*Financial stress—tangible social support*								
No stress—high support	51 792	61.5	24 856	64.5	26 936	58.9	83.53	<0.001
Medium stress—high support	12 742	15.1	4689	12.2	8053	17.6	888.13	<0.001
High stress—high support	4900	65.8	1700	4.4	3200	7.0	459.18	<0.001
No stress—low support	6902	8.2	3843	10.0	3059	6.7	89.06	<0.001
Medium stress—low support	2724	3.2	1215	3.2	1509	3.3	31.73	<0.001
High stress—low support	1788	2.1	792	2.1	996	2.2	23.28	<0.001
Missing data	3415	4.1	1464	3.8	1951	4.3	69.45	<0.001

In the group with high tangible social support, high financial stress increased the likelihood of low psychological well-being by nearly three times among men and by more than two times among women compared with no financial stress (Table 2). By contrast, in the group with low tangible social support, high financial stress increased the likelihood of low psychological well-being by nearly seven times among men and by nearly six times among women compared with no financial stress and high tangible social support. In the group with high tangible social support, high financial stress increased the likelihood of many psychosomatic symptoms by four times in men and by nearly three times in women compared with no financial stress (Table 3). By contrast, in the group with low tangible social support, high financial stress increased the likelihood of many psychosomatic symptoms by more than seven times in men and by more than five times in women compared with no financial stress and high tangible social support. The adjustment for educational level was not performed in the analyses because of the large number of missing cases for this variable. However, a re-run of the analyses of the data in Tables 2 and 3 with the inclusion of educational level did not alter the results in any significant way, although the odds ratios (ORs) for each subgroup increased slightly in both models.

**Table 2:** **Binary logistic regression of financial stress – social support model in relation to low psychological well-being in men and women**

	**Low psychological well-being**					
	**Men**			**Women**		
	**N 36 444** ^**a**^	**%**	**OR (95% CI)** ^**b, c**^	**N 42 648** ^**a**^	**%**	**OR (95% CI)** ^**b, d**^
		*** ^**e**^			*** ^**e**^	
No financial stress—high tangible social support	1935^f^	8.1^g^	1 *(ref)*	3535^f^	13.9^g^	1 *(ref)*
Medium financial stress—high tangible social support	716	16.0	1.65 (1.50–1.82)***	1707	22.5	1.41 (1.32–1.51)***
High financial stress—high tangible social support	440	26.9	2.94 (2.59–3.32)***	1104	35.8	2.37 (2.17–2.58)***
No financial stress—low tangible social support	533	14.9	2.06 (1.85–2.29)***	716	25.5	2.51 (2.28–2.76)***
Medium financial stress—low tangible social support	286	25.5	3.24 (2.79–3.75)***	509	37.1	3.23 (2.86–3.65)***
High financial stress—low tangible social support	339	45.3	6.73 (5.75–7.89)***	542	57.0	5.89 (5.13–6.77)***

The data are presented as numbers and percentages of participants with low psychological well-being within each subgroup, odds ratios (ORs), and 95% confidence intervals (CIs).

***p ≤ 0.001.

^a^Total number of participants included in the analysis.

^b^Adjusted for age, country of birth, employment status, other chronic disease, and year of survey.

^c^Nagelkerke *R* ^*2*^ = 0.130.

^d^Nagelkerke *R* ^*2*^ = 0.145.

^e^χ^2^ test of differences between the subgroups within each sex.

^f^ Number of participants within each subgroup who reported low psychological well-being.

^g^Percentage of participants within each subgroup who reported low psychological well-being.

**Table 3:** **Binary logistic regression of financial stress – tangible social support model in relation to having many psychosomatic symptoms in men and women**

	**Many psychosomatic symptoms**					
	**Men**			**Women**		
	**N 36 346** ^**a**^	**%**	**OR (95% CI)** ^**b, c**^	**N 42 514** ^**a**^	**%**	**OR (95% CI)** ^**b, d**^
		*** ^**e**^			*** ^**e**^	
No financial stress—high tangible social support	1590^f^	6.7^g^	1 *(ref)*	3957^f^	15.6^g^	1 *(ref)*
Medium financial stress—high tangible social support	594	13.3	1.88 (1.69–2.09)***	1879	24.8	1.62 (1.51–1.73)***
High financial stress—high tangible social support	419	25.6	4.00 (3.50–4.58)***	1223	39.7	2.95 (2.70–3.22)***
No financial stress—low tangible social support	459	12.8	1.85 (1.65–2.08 )***	814	29.1	2.00 (1.82–2.20)***
Medium financial stress—low tangible social support	237	21.3	3.24 (2.75–3.82)***	553	40.3	3.00 (2.65–3.39)***
High financial stress—low tangible social support	307	41.0	7.45 (6.28–8.85)***	541	57.0	5.49 (4.74–6.35)***

The data are presented as numbers and percentages of participants having many psychosomatic symptoms within each subgroup, odds ratios (ORs), and 95% confidence intervals (CIs).

***p ≤ 0.001.

^a^Total number of participants included in the analysis.

^b^Adjusted for age, country of birth, employment status, other chronic disease, and year of survey.

^c^Nagelkerke *R* ^*2*^ = 0.212.

^d^Nagelkerke *R* ^*2*^ = 0.227.

^e^χ^2^ test of differences between the subgroups within each sex.

^f^Number of participants within each subgroup who reported having many psychosomatic symptoms.

^g^Percentage of participants within each subgroup who reported having many psychosomatic symptoms.

**Figure 1:**
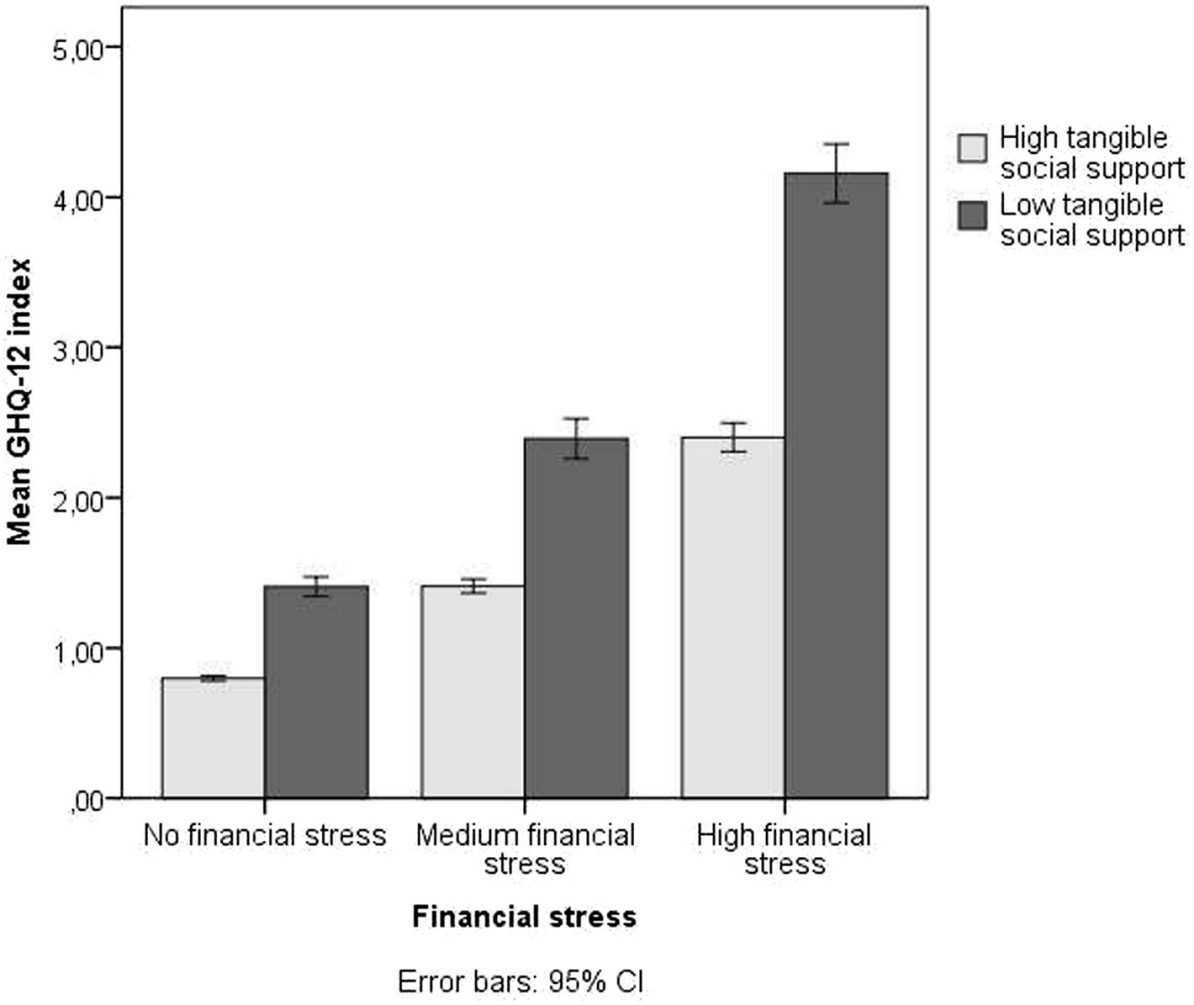
**Financial stress and tangible social support in relation to the GHQ-12 index.**

The GLM was adjusted for age, country of birth, employment status, other chronic disease, and year of survey. There were significant interaction effects between financial stress and tangible social support in relation to low psychological well-being; the strongest effect was in men (Table 4). These findings suggest that the effect of tangible social support was stronger in the presence of higher financial stress, as also shown in Figure 1. The interaction effects between financial stress and tangible social support in relation to psychosomatic symptoms were noticeably weaker in men and were not significant in women.

**Table 4:** **General linear models showing interaction effects between financial stress and tangible social support in relation to low psychological well-being and psychosomatic symptoms**

	**Low psychological well-being** ^**a**^						**Psychosomatic symptoms** ^**a**^					
	**Men**			**Women**			**Men**			**Women**		
	**df**	**F**	**p**	**df**	**F**	**p**	**df**	**F**	**p**	**df**	**F**	**p**
1. Financial stress	1, 35 298	317.64	<0.001	1, 41 285	179.06	<0.001	1, 35 217	91.04	<0.001	1, 41 185	39.54	<0.001
2. Tangible social support	1, 35 298	250.50	<0.001	1, 41 285	615.60	<0.001	1, 35 217	427.03	<0.001	1, 41 185	598.46	<0.001
1 × 2	1, 35 298	166.08	<0.001	1, 41 285	8.57	<0.001	1, 35 217	7.10	0.008	1, 41 185	0.46	0.499

^a^Adjusted for age, country of birth, employment status, other chronic disease, and year of survey.

## Discussion

The present study investigated the buffering effect of tangible social support on financial stress in relation to psychological well-being and psychosomatic symptoms in a large sample of the adult general population in Sweden. In the group with high tangible social support, high financial stress increased the likelihood of low psychological well-being by two to three times and the likelihood of many psychosomatic symptoms by three to four times. However, in the group with low tangible social support, high financial stress increased the likelihood of both low psychological well-being and many psychosomatic symptoms by six to seven times when compared with no financial stress and high tangible social support. The associations between financial stress, low tangible social support, and ill health were more prominent among men. Consistent with the buffering hypothesis, there were significant interactions between financial stress and tangible social support in relation to psychological well-being. However, the analyses of psychosomatic symptoms showed weaker interaction effects in men and no effects in women. These findings suggest that tangible social support has its strongest buffering effect on psychological well-being at high levels of financial stress. However, tangible social support has a weaker effect on the relationship between financial stress and psychosomatic symptoms, particularly in women. Financial stress had a substantial impact on emotional and physical well-being even when tangible social support was available.

The present findings partly support the suggestion that the buffering effect on a specific life stress is prominent only if the social support factor is aimed at alleviating the specific stress [14,15]. Similar buffering effects of tangible social support have been found in previous studies of financial stress in relation to psychological well-being [16] and alcohol consumption [19]. However, a study by Krause et al. investigating chronic financial strain in relation to depressive symptoms found no buffering effect of tangible social support [28]. That study focused on a population comprising solely elderly people (aged >65 years). Although the present study involved a random sample of the population aged 18–84 years, the differences in response rates caused an imbalance in the data toward a preponderance of older participants. This suggests that differences in findings between the Krause et al. study and the present study might not be explained by population factors. Rather, the difference in outcome measures is the most plausible explanation, as the more severe condition of depression might be less influenced by tangible social support and is not comparable to the less severe, general measures of psychological well-being and psychosomatic symptoms in the present study.

The buffering effect of tangible social support may be explained by several possible mechanisms. The perception that others can and will provide necessary resources may redefine the potential for harm and prevent the situation from being appraised as highly stressful [14]. The reception of support signals that others care for and value the distressed person, boosting his or her sense of mattering and self-esteem [7]. The perception of available tangible support may thus intervene between the experience of financial stress and the pathological outcome by reducing the stress reaction and its harmful physiological processes. Tangible social support may also provide a solution to the problem associated with the stress factor by providing support and tangible help in the event of personal problems or crises, thereby reducing the stress reaction. Although the instrument used in the present study did not include direct questions about receiving financial assistance, the types of assistance described (whether the person could expect aid in personal crises, receive help with daily chores if they became sick, and receive help if they were moving) may be crucial to the easing or solving of financial problems.

The question of whether altering one’s social networks may improve physical health is very important [29]. From a health-care perspective, the prevention of ill health and promotion of good health may influence both health-care costs and quality of life. The answer to this question may provide important insights into and tools for pursuing basic social psychological questions, such as how the characteristics of our social networks influence our cognitive, behavioural, and physiological functions [29].

### Limitations

There are several limitations in the present study. The overall response rate of 61.4% was not optimal. There were also differences in response rates between the subgroups within the sample; for example, males, younger individuals, those with a lower educational level, and individuals born outside Sweden all had lower response rates [26,27]. However, the statistical analyses controlled for potential confounding factors such as age, country of birth, employment status, other chronic disease, and year of survey. Because of the number of missing answers about educational level (about 13%), we chose to exclude it as a control variable from the analyses. However, a re-run of the analyses with educational level included revealed no major alterations of the results. Moreover, the imbalance in the data toward a preponderance of older participants may have influenced our results because it has been suggested that financial stress and social support may be particularly important for older people [28,29]. Our results may therefore be less generalisable to younger populations. Because of the anonymous study design, it was not possible to do a thorough non-response analysis to investigate this.

The cross-sectional design restricts the conclusions that can be drawn about cause and effect. Although our results show strong associations between financial stress, tangible social support, and ill health, the directions of the associations are unknown. Low psychological well-being and many psychosomatic symptoms might, for example, be related to higher rates of sick leave [30] or early retirement [31], life situations that are closely associated with lower income and higher risk of financial stress.

Moreover, the question in the financial stress measure regarding the ability to raise money did not specify between raising money from personal savings or from their support network, which could have created collinearity with the tangible support measure. Also, the social support question regarding “persons around you who would give you support in the event of personal problems or crises” may not necessarily be interpreted by the participants as getting tangible social support. However, we still deemed tangible social support to be the definition that best corresponded to the social support measure.

Another limitation regards the dichotomisation of the measures which might reduce the specificity of the data. These dichotomisations were necessary in order to create a balanced financial stress–tangible social support model, which was analysed by binary logistic regression. However, we complemented these analyses with a general linear model, using the non-dichotomised indices, which showed similar findings. The procedure with complementary statistical approaches can help to overcome shortcomings with the individual statistical methods and help to eliminate scaling artefacts.

Although we adjusted the analyses for confounding from chronic disease, we were not able to specify whether the chronic disease concerned a mental disorder or a physical disorder. It has been suggested that prior mental disorders can be a major risk factor for future stress generation [32,33]. Moreover, financial stress and ill health are related to several confounding demographic and psychosocial factors that were not controlled for in the present study and that might partly explain the findings, i.e., occupational class, income, marital status, long-term unemployment, and trade conditions in society. Since societal factors such as trade conditions and unemployment rates might have changed between the two surveys, this could have affected the results. Therefore, we adjusted all analyses for survey year.

However, the limitations may be balanced by the statistical power. Overall, there were 84 263 respondents, and the smallest subgroup of the financial stress — tangible social support model included 792 men and 996 women. These subgroup sizes equal the total population samples of many other studies and counteract the risk of random findings that accompany small sample designs. The strong associations between financial stress and tangible social support in relation to ill health are particularly interesting given that the study was set in Sweden, a highly egalitarian country with well-developed social security and social-welfare programs. Thus, poverty and financial stress might not be as devastating or life-threatening in Sweden as in countries with less developed social-welfare systems.

## Conclusions

The question of whether altering our social networks may improve physical and mental health is important for the prevention of ill health, and the answer to this question may help reduce health-care costs and improve quality of life. Future research should focus on carefully defining the dimensions of social support that meet the demands of a specific life stressor, preferably in large generalisable population studies. The next step in the social support research field would be to use interdisciplinary methods to investigate further the physiological mechanisms behind the stress-buffering effects, such as the neuroendocrine and neurological pathways.

## Abbreviations

GHQ-12: General Health Questionnaire
GLM: General Linear Models
SD: Standard Deviation
OR: Odds Ratio
CI: Confidence Interval
